# Identifying an outbreak of a novel swine disease using test requests for porcine reproductive and respiratory syndrome as a syndromic surveillance tool

**DOI:** 10.1186/1746-6148-8-192

**Published:** 2012-10-16

**Authors:** Terri L O’Sullivan, Robert M Friendship, David L Pearl, Beverly McEwen, Catherine E Dewey

**Affiliations:** 1 Department of Population Medicine, Ontario Veterinary College, University of Guelph, Guelph, Ontario, N1G 2W1, Canada; 2 Animal Health Laboratory, Laboratory Services Division, University of Guelph, Guelph, Ontario, N1H, Canada

**Keywords:** Swine, Disease surveillance, Laboratory data, Syndromic surveillance, Diagnostic test requests, Test results

## Abstract

**Background:**

Animal disease monitoring and surveillance are crucial for ensuring the health of animals, humans and the environment. Many studies have investigated the utility of monitoring syndromes associated with data from veterinary laboratory submissions, but no research has focused on how negative test results from a veterinary diagnostic laboratory data can be used to improve our knowledge of disease outbreaks. For example, if a diagnostic laboratory was seeing a disproportionate number of negative test results for a known disease could this information be an indication of a novel disease outbreak? The objective of this study was to determine the association between the porcine circovirus associated disease (PCVAD) outbreak in Ontario 2004–2006 and the results of porcine reproductive and respiratory syndrome virus (PPRSV) enzyme-linked immunosorbent assay (ELISA) and the results of PRRSV polymerase chain reaction (PCR) diagnostic tests requested by veterinarians.

**Results:**

Retrospective data were collected from the Animal Health Laboratory (AHL) at the University of Guelph, Guelph, Ontario Canada and were comprised of weekly counts of PRRSV ELISA and PRRSV PCR diagnostic tests requested by swine practitioners from 2000–2007. The results of the PRRSV ELISA and PRRSV PCRs were analysed separately in two models using logistic regression with the dependent variables being: the weekly probability of PRRSV ELISA positivity, and the weekly probability of PRRSV PCR positivity, respectively. The weekly probability of PRRSV PCR positivity decreased during the PVCAD outbreak (OR=0.66, *P*=0.01). The weekly probability of PRRSV ELISA positivity was not associated with the PCVAD outbreak.

**Conclusions:**

The results of this study showed that during the PCVAD outbreak in Ontario from December 2004-May 2006, the probability of a positive PRRSV PCR at the AHL decreased. We conclude that when a decrease in test positivity occurs for a known disease, it may suggest that a new disease agent is emerging in the population. Hence, monitoring the test results of commonly used first-order tests for a known disease (e.g. PRRSV) has the potential to be a unique form of syndromic data for the timely identification of novel disease outbreaks in swine populations.

## Background

The information captured by veterinary diagnostic laboratories generates an immense database of animal health information and has contributed significantly to the collective knowledge of animal diseases. In addition to playing a role in determining disease etiology, the data are crucial in providing essential health information for disease monitoring and passive disease surveillance systems of livestock industries worldwide [1-3]. In response to the need for improving and implementing coordinated disease surveillance for Canadian livestock sectors, the Canadian Animal Health Surveillance Network (CAHSN) was established and veterinary diagnostic laboratory data contribute significantly to the network [4]. The use of laboratory data for passive disease surveillance is limited by its lack of timeliness in identifying disease outbreaks, re-emerging diseases, or novel pathogens [2,4,5]. The primary reason for this lack of timeliness is the delay that occurs between the time of submission to the point when the final test results are available. Current research is actively investigating novel methods to improve the use of laboratory-derived data in disease monitoring and surveillance capacities [6-8].

To the authors’ knowledge, no research has documented the association between the proportion of positive or negative test results for known swine diseases and the occurrence of a novel swine disease outbreak. For example, if a diagnostic laboratory experiences a disproportionate number of negative test results i.e., more tests with negative results than expected, could this information indicate that practicing veterinarians are seeing an unknown disease that represents a novel or re-emerging disease outbreak?

Porcine reproductive and respiratory syndrome virus (PRRSV) has challenged the global swine industry for years; and despite herd, region, or country eradication programs, remains a significant swine disease challenge [9,10]. PRRSV infection presents with numerous and varied clinical signs in multiple age groups of pigs. This may explain why astute swine veterinarians regularly monitor client herds for PRRSV, and why swine disease outbreak investigations start with an examination for the presence of PRRSV [10]. From a disease monitoring and surveillance point of view this raises the following question. Would monitoring the results of PRRSV first-order (screening) tests requested by veterinarians at a diagnostic laboratory be useful for signalling a novel swine disease outbreak?

Numerous diagnostic tests are available for detection of PRRSV antigens or antibodies. However, the PRRSV enzyme-linked immunosorbent assay (ELISA) and the PRRSV polymerase chain reaction (PCR) are common first-order tests requested by swine veterinarians [10]. A first-order PRRSV test is defined as a routinely used screening test typically selected for the initial, and rapid, investigation of a swine herd disease problem where PRRSV is suspected [10]. The commercially available ELISA (HerdChek® 2XR/3XR PRRS ELISA, IDEXX Laboratories Inc., Westbrook, Maine. USA), the gold standard for antibody detection and has a rapid turn-around time [11]. PRRSV PCRs used to detect viral nucleic acid in tissues, serum, and semen, also have a rapid turnaround time [12].

In the late fall of 2004, an outbreak of porcine circovirus associated disease (PCVAD) caused by a highly pathogenic variant of porcine circovirus type-2, (PCV-2) occurred in Ontario, Canada [13]. The outbreak spread rapidly, was associated with high mortality, and was difficult to control until a highly efficacious vaccine became available by special licence on March 1, 2006 [13,14]. The industry also experienced a concurrent outbreak of a novel strain of PRRSV October 2004 to March 2005 [15].

Infection with PCV-2 causes a wide range of systemic clinical signs similar to some clinical signs associated with PRRSV infection. Severe weight loss (wasting), failure-to-thrive, and pneumonia are clinical signs common to both PRRS and PCV-2 [10,13]. Infection with PCV-2 is considered an important differential diagnosis for PRRS [10]. Hence, it was hypothesized that the probability of positive PRRSV ELISAs and PRRSV PCRs requested by swine practitioners at the Animal Health Laboratory (AHL), University of Guelph, Guelph, Ontario, Canada, would decrease during the PCVAD outbreak. The objectives of this study were to determine how the PCVAD outbreak in Ontario, Canada, from December 2004 - May 2006 influenced the number of positive results of PRRSV ELISAs and PRRSV PCRs requested by practicing veterinarians at the AHL after taking into consideration season, year, and a concurrent PRRSV outbreak.

## Methods

### Data source and variables

Retrospective AHL diagnostic test data requested by swine veterinarians were compiled from January 1, 2000 to April 30, 2007 and collapsed into weekly counts. The AHL provides services for researchers as well as for private practitioners. For the purposes of this study, diagnostic test data associated with research cases were excluded as were tests used for herd monitoring and those associated with semen specimens. First-order PRRSV tests were considered for potential inclusion in the analysis and included the PRRSV ELISA and the PRRSV PCRs offered by the AHL during the study period. Diagnostic tests associated with follow-up requests, such as gene typing or sequencing, were not considered in the current study, as they have a slower turn-around time and do not represent first-order tests.

The PRRSV ELISA offered at the AHL laboratory did not change with respect to its test performance during the study period. In 2002, AHL’s PRRSV ELISA was modified to include two recombinant protein preparations representing United States and European strains, but equivalent test performance and cut-off were maintained. [11]. The most predominant PRRSV PCR test offered at the AHL during the study period was available from June 29, 1998 until December 4, 2006, but it did experience a minor improvement in test performance [16]. The most notable change occurred December 4, 2006 with the introduction of the PRRSV PCR-Tetracore test. This test change occurred after the end of the PCVAD outbreak.

The first-order PRRSV diagnostic tests selected for the analyses were the PRRSV ELISA and PCRs requested from January 1, 2000 until April 30, 2007. These two tests were considered unique and were analysed separately. The weekly count of positive PRRSV ELISAs and the total weekly count of requested PRRSV ELISAs were determined and used to represent the dependent variable, weekly probability of positive PRRSV ELISA results. Similarly, the weekly count of positive PRRSV PCRs and the total weekly count of requested PRRSV PCRs were determined and used to represent the dependent variable, weekly probability of positive PRRSV PCR results.

Two dichotomous variables were generated and coded (1= outbreak, 0= no outbreak) to represent the two disease outbreaks experienced by the Ontario swine industry during the study period: the PCVAD outbreak that occurred in Ontario from 2004–2006 [13] and the PRRSV outbreak that occurred in 2004–2005 [15]. The PRRSV outbreak was considered to be a confounder *a priori*. Season was also considered to be a confounder *a priori* and was examined as a categorical variable representing Winter (Dec-Feb), Spring (Mar-May), Summer (June-Aug), and Fall (Sept-Nov). Fall was the referent season in the model. Year was modelled as a dummy variable with 2000 the referent year in the model.

### Statistical analysis

All statistical analyses were conducted in Stata 11(Stata Corp., College Station, Texas, USA).

### Descriptive statistics and univariable associations

The PRRSV ELISA and PCR results were analysed separately. The two dependent variables of interest were the “weekly probability of PRRSV ELISA positivity” and the “weekly probability of PRRSV PCR positivity”. The dependent variable “weekly probability of ELISA positivity” was created by taking the number of positive ELISAs per week and dividing that by the total number of ELISAs requested that week. Similarly, the dependent variable “weekly probability of PCR positivity” was created by taking the number of positive PCRs per week and dividing that by the total number of PCRs requested that week. The dependent variables were examined by graphing time series plots to observe the trend of the variables over time and by examining distribution plots. Standard descriptive statistics were calculated.

All of the above covariates were then evaluated for statistical significance with the dependent variable “weekly probability PRRSV ELISA positivity” using logistic regression in a generalized linear model (GLM) framework that used maximum likelihood (ML) estimation [17]. A logit link function and a binomial distribution were used with the total weekly count of PRRSV ELISA tests as the denominator. A liberal *P*-value of ≤0.2 was set to be significant to pre-screen the variables. Univariable associations between the above covariates were then evaluated for statistical significance with the dependent variable “weekly probability of PRRSV PCR positivity” as described for the PRRSV ELISA positivity data.

### Model A: logistic regression using a GLM approach

The independent variables previously identified by univariable associations as having a liberal significance of *P*≤0.2 with PRRSV ELISA positivity were put into a multivariable GLM model (Model A). Interactions between year and season, and between the PCVAD outbreak and the PRRS outbreak were investigated for significance (*P*≤0.05). Then, by using likelihood ratio tests, a backwards elimination process was employed to identify the final model retaining the significant (*P*≤0.05) main effects variables and interaction terms [18]. As each variable was removed from the model, the coefficients of the other variables were examined for evidence of confounding as indicated by a change of 20% in any of the remaining coefficients. If the confounding criteria were met, then the confounding variable was forced back into the model. None of the variables were considered intervening variables.

Model fit using the Pearson Chi-square goodness-of-fit test was performed. Graphical visualization of the scatter-plot of the Pearson residuals against the predicted outcome was used to assess outliers. Subsequently, a partial autocorrelation function (PAF) plot was used to assess whether any autocorrelation remained in the Pearson residuals [18,19].

### Model B: logistic regression using a GLM approach

The same GLM model-building process and model diagnostics used for Model A were repeated for the second model (Model B) using the PRRSV PCR positive results as the dependent variable. A logit link function and a binomial distribution were used with the total weekly count of PRRSV PCR tests used as the denominator.

## Results

### Descriptive statistics and univariable associations

**Figure 1:**
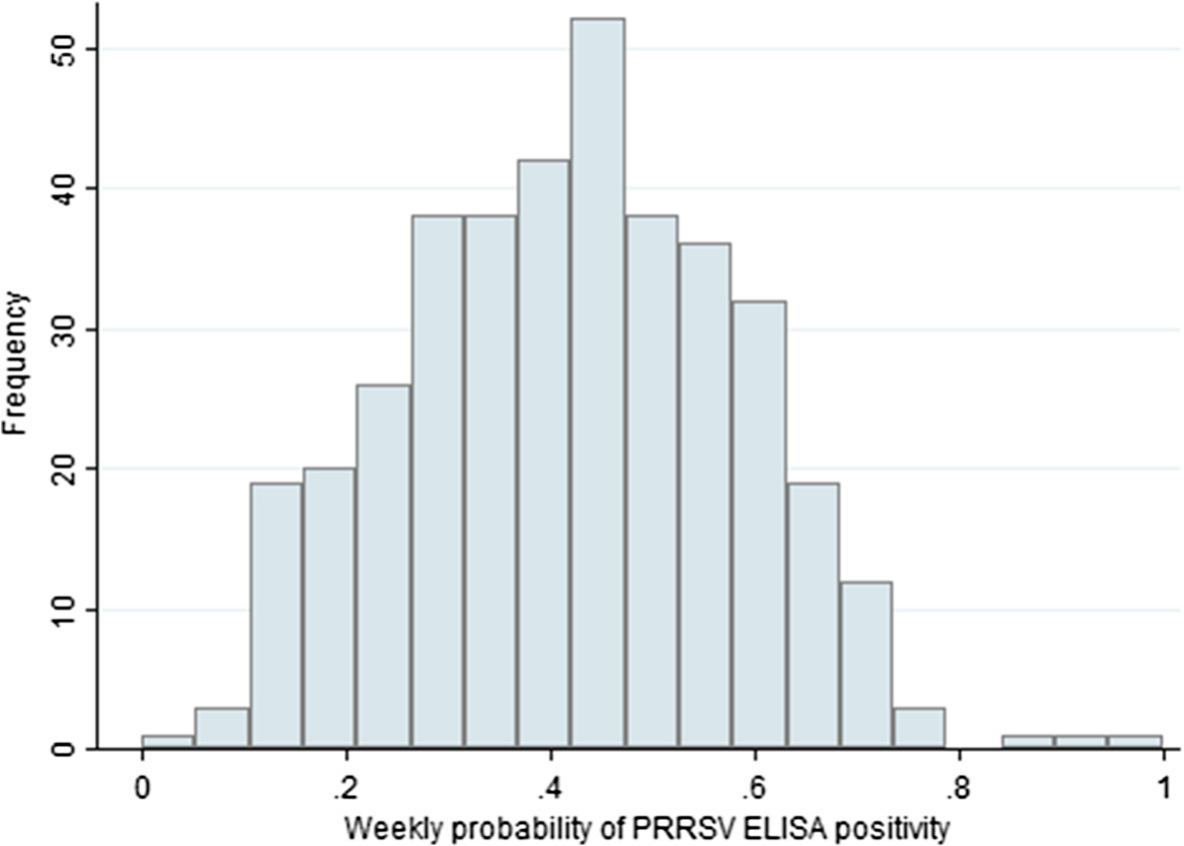
Distribution of the weekly probability of PRRSV ELISA positivity at the Animal Health Laboratory from January 1, 2000 to April 30, 2007.

**Figure 2:**
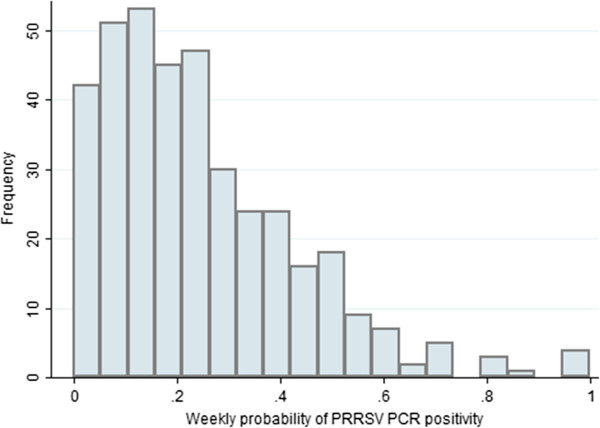
Distribution of the weekly probability of PRRSV PCR positivity at the Animal Health Laboratory from January 1, 2000 to April 30, 2007.

**Figure 3:**
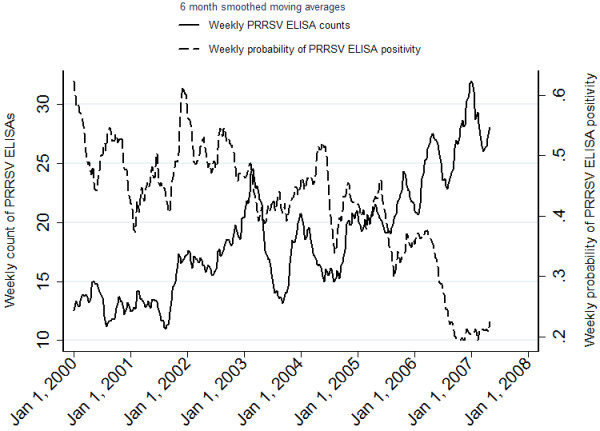
Time series plot of the weekly count of PRRSV ELISAs requested and the weekly probability of PRRSV ELISA positivity at the Animal Health Laboratory from January 1, 2000 to April 30, 2007.

**Figure 4:**
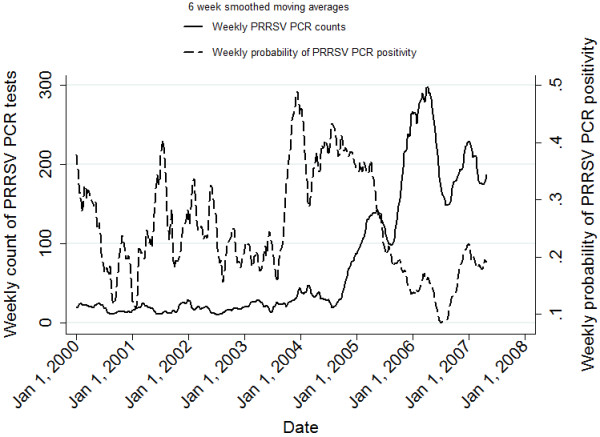
Time series plot of the weekly count of PRRSV PCR tests requested and the weekly probability of PRRSV PCR positivity at the Animal Health Laboratory from January 1, 2000 to April 30, 2007.

A total of 7,092 PRRSV ELISA and 28,601 PRRSV PCRs were requested at the AHL from January 1, 2000 - April 30, 2007. The means of the weekly count of PRRSV ELISA and PRRSV PCRs were 18.6 (SD=7.0) and 74.9 (SD=92.2), respectively. The total number of observed weeks was 382 and the overall mean of the weekly probability of positive PRRSV ELISA results was 42.1% (SD=16.2). The overall mean of the weekly probability of positive PRRSV PCR results was 24.8% (SD=19.2). The distribution of the weekly probability of PRRSV ELISA positivity and the weekly probability of PRRSV PCR positivity are presented in Figures 1 and 2, respectively. The time series plots of the weekly probabilities of PRRSV ELISA positivity and PRRSV PCR positivity are presented in Figures 3 and 4, respectively.

The independent variables considered for the full main effects multivariable GLM logistic regression model and their univariable associations with the dependent variable “weekly probability of PRRSV ELISA positivity,” are shown in Table 1. The independent variables considered for the full main effects multivariable GLM logistic regression model and their univariable associations with the dependent variable “weekly probability of PRRSV PCR positivity,” are shown in Table 2.

**Table 1:** **Univariable associations**^**a **^**between the weekly probability of PRRSV ELISA positivity at the Animal Health Laboratory from January 1, 2000 to April 30, 2007 and the PCVAD outbreak of the Ontario swine industry, a PRRSV outbreak, season, and year**

**Variable**		**n**	**OR**^**b**^	**95% CI**	***P *-value**
PCVAD outbreak			0.87	0.77 - 0.97	0.02
PRRSV outbreak^c^			1.19	1.04 - 1.37	0.01
Season^c^	Fall	91	Referent	-	-
	Spring	101	1.08	0.95 - 1.24	0.25
	Summer	91	1.05	0.91 - 1.21	0.50
	Winter	99	1.05	0.92 - 1.20	0.77
Year	2000	-	Referent	-	-
	2001	52	0.89	0.72 - 1.10	0.28
	2002	52	0.97	0.79 - 1.18	0.75
	2003	52	0.77	0.63 - 0.94	0.01
	2004	52	0.78	0.64 - 0.95	0.01
	2005	52	0.60	0.49 - 0.73	<0.001
	2006	52	0.37	0.31 - 0.45	<0.001
	2007	18	0.25	0.19 - 0.32	<0.001

a. Logistic regression using generalized linear model ML estimation with the logit link, binomial distribution and total number of PRRSV ELISA tests ordered representing the denominator.

b. Odds ratio.

c. Suspected *a priori* may be a confounding variable.

**Table 2:** **Univariable associations**^**a **^**between the weekly probability of PRRSV PCR positivity at the Animal Health Laboratory from January 1, 2000 to April 30, 2007 and the PCVAD outbreak of the Ontario swine industry, a PRRSV outbreak , season, and year**

**Variable**		**n**	**OR**^**b**^	**95% CI**	***P *-value**
PCVAD outbreak			1.08	1.02 - 1.14	0.01
PRRSV outbreak^c^			2.41	2.22 - 2.62	<0.001
Season^c^	Fall	91	Referent	-	-
	Spring	101	1.12	1.04 - 1.22	0.005
	Summer	91	0.76	0.69 - 0.84	<0.001
	Winter	99	1.05	0.97 - 1.14	0.22
Year	2000	52	Referent	-	-
	2001	52	0.79	0.63 - 0.99	0.04
	2002	52	1.05	0.84 - 1.31	0.68
	2003	52	0.99	0.81 - 1.22	0.93
	2004	52	1.79	1.50 - 2.15	<0.001
	2005	52	1.14	0.97 - 1.35	0.11
	2006	52	0.57	0.48 - 0.67	<0.001
	2007	18	0.88	0.73 - 1.05	0.14

a. Logistic regression using generalized linear model ML estimation with the logit link, binomial distribution and total number of PRRSV PCR tests ordered representing the denominator.

b. Odds ratio.

c. Suspected *a priori* may be a confounding variable.

### Final Model A: weekly probability of PRRSV ELISA positivity

**Figure 5:**
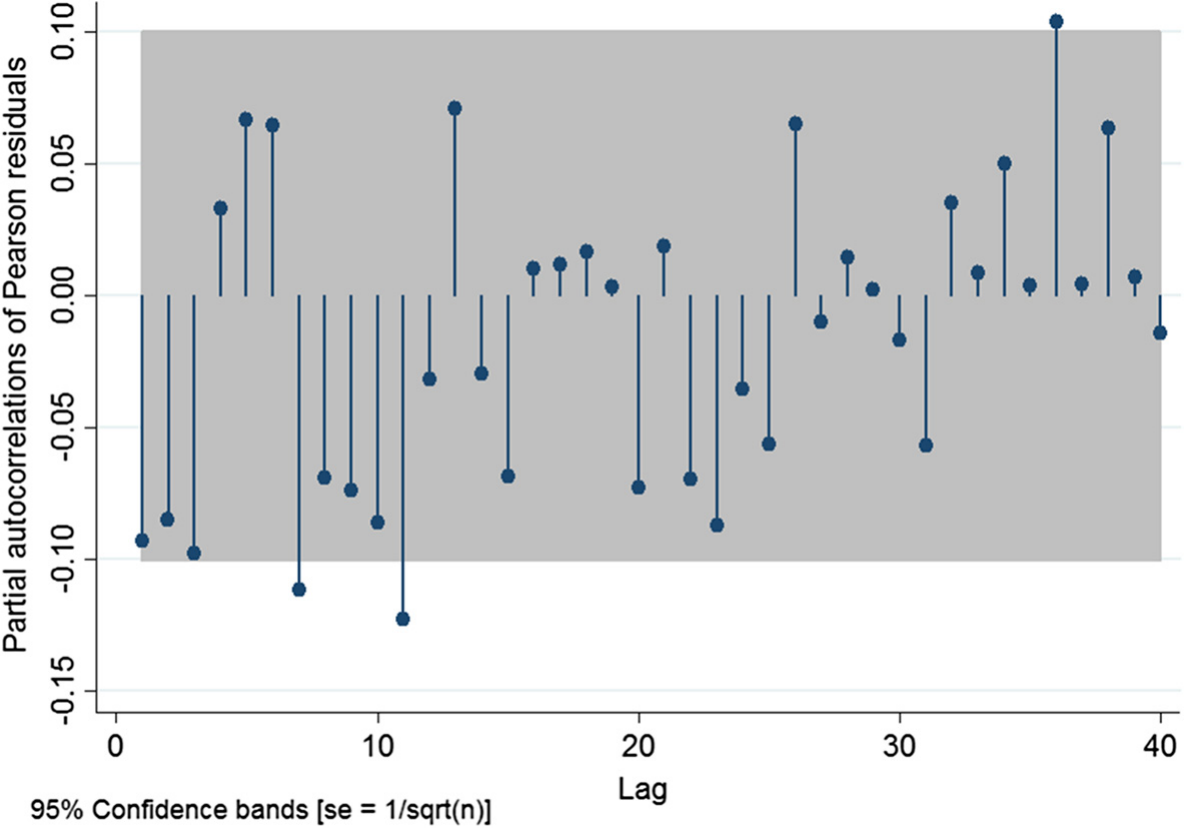
Partial autocorrelation function plot of the Pearson residuals for Model A.

The final multivariable GLM logistic regression model including all significant main-effects terms and interaction terms, is shown in Table 3. After controlling for season, the PRRSV outbreak, and year, and the season-year interaction term, the weekly probability of PRRSV ELISA positivity was not associated with the PVCAD outbreak. The interaction between season and year was the only significant interaction term. Model diagnostics showed adequate model fit. No serial correlation was identified in the Pearson residuals of the final model (Figure 5).

**Table 3:** **The association**^**a**^**between the weekly probability of PRRSV ELISA positivity and the PCVAD outbreak of the Ontario swine industry after controlling for a PRRSV outbreak, season and year using data from the Animal Health Laboratory from January 2000 and April 2007**

**Variable**		**n**	**OR**^**b**^	**95% CI**	***P *-value**
PCVAD outbreak			1.33	0.97 - 1.83	0.08
PRRSV outbreak^c^			0.83	0.56 - 1.24	0.36
Season^c^	Fall	91	Referent	-	-
	Spring	101	0.86	0.56 - 1.28	0.43
	Summer	91	1.01	0.66 - 1.54	0.98
	Winter	99	1.12	0.73 - 1.72	0.62
Year	2000	52	Referent	-	-
	2001	52	0.92	0.61 - 1.38	0.68
	2002	52	1.06	0.72 - 1.57	0.76
	2003	52	0.66	0.44 - 0.99	0.047
	2004	52	0.86	0.49 - 1.50	0.59
	**2005**	**52**	**0.36**	**0.22 - 0.59**	**<0.001**
	**2006**	**52**	**0.22**	**0.15 - 0.34**	**<0.001**
	**2007**	**18**	**0.22**	**0.15 - 0.34**	**<0.001**
Year*Season interaction^d^	2001*spring	0.84	1.15	0.64 - 2.07	0.64
	2001*summer	0.88	0.58	0.46 - 1.54	0.58
	2001*winter	1.10	0.66	0.49 - 1.58	0.66
	2002*spring	0.89	0.73	0.63 - 1.92	0.73
	2002*summer	0.69	0.69	0.51 - 1.57	0.69
	2002*winter	1.17	0.19	0.39 - 1.21	0.19
	2003*spring	1.13	0.58	0.67 - 2.05	0.58
	2003*summer	1.32	0.68	0.63 - 2.04	0.68
	2003*winter	1.65	0.33	0.75 - 2.33	0.33
	2004*spring	0.88	0.08	0.95 - 2.87	0.08
	2004*summer	0.76	0.65	0.50 - 1.55	0.65
	2004*winter	**1.70**	0.41	0.40 - 1.45	0.41
	2005*spring	1.09	**0.05**	**0.99 - 2.90**	**0.05**
	2005*summer	1.46	0.75	0.63 - 1.90	0.75
	2005*winter	**2.24**	0.23	0.79 - 2.72	0.23
	2006*spring	1.50	**0.007**	**1.25 - 4.00**	**0.007**
	2006*summer	1.25	0.16	0.86 - 2.63	0.16
	2006*winter	1.26	0.47	0.69 - 2.26	0.47
	2007*spring	0.84	0.42	0.69 - 2.31	0.42

a. Logistic regression using generalized linear model ML estimation with the logit link, binomial distribution and total number of PRRSV PCR tests ordered representing the denominator.

b. Odds ratio.

c. Suspected *a priori* may be a confounding variable.

d. Interaction term.

bold text – significant variables.

### Final Model B: weekly probability of PRRSV PCR positivity

**Figure 6:**
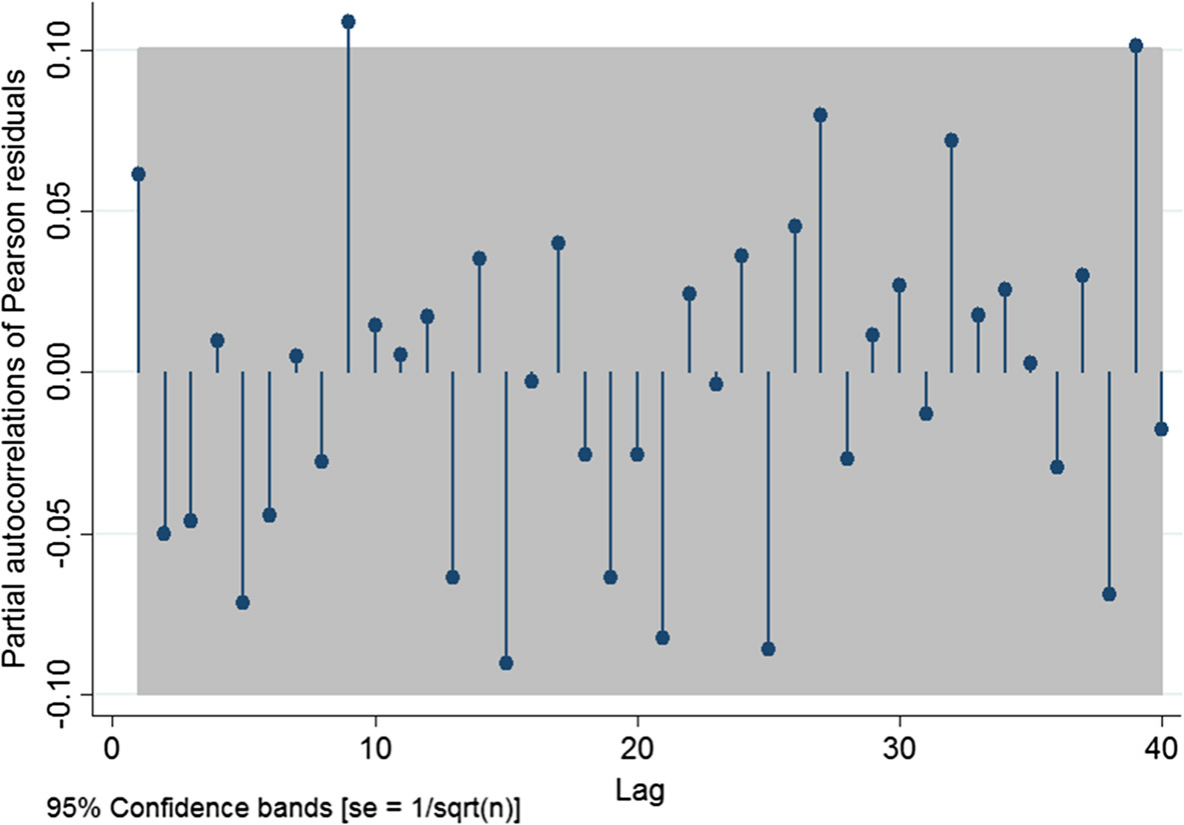
Partial autocorrelation function plot of the Pearson residuals for Model B.

The final multivariable logistic regression model, including all significant main-effects terms and interaction terms, is displayed in Table 4. After controlling for season, the PRRSV outbreak, and year, and the season-year interaction term, the weekly probability of PRRSV PCR positivity decreased during the PCVAD outbreak (OR=0.66, *P=*0.01). Model diagnostics showed adequate model fit. No serial correlation was identified in the residuals of the final model (Figure 6).

**Table 4:** **The association**^**a**^**between the weekly probability of PRRSV PCR positivity and the PCVAD outbreak of the Ontario swine industry after controlling for a PRRSV outbreak, season and year using data from the Animal Health Laboratory from January 2000 and April 2007**

**Variable**		**n**	**OR**^**b**^	**95% CI**	***P*-value**
**PCVAD outbreak**			**0.66**	**0.58 - 0 .75**	**0.01**
**PRRSV outbreak**^**c**^			**2.53**	**2.14 - 2.97**	**<0.001**
Season^c^	Fall	91	Referent	-	
	Spring	101	0.92	0.60 - 1.43	0.72
	**Summer**	**91**	**0.57**	**0.35 - 0.93**	**0.02**
	Winter	99	0.85	0.55 - 1.33	0.49
Year	2000	52	Referent	-	-
	2001	52	0.81	0.51 - 1.30	0.38
	2002	52	0.95	0.60 - 1.52	0.84
	2003	52	0.90	0.60 - 1.35	0.61
	**2004**	**52**	**0.66**	**0.44 - 0.99**	**0.04**
	2005	52	1.04	0.71 - 1.50	0.85
	**2006**	**52**	**0.49**	**0.35 - 0.70**	**<0.001**
	2007	18	0.85	0.62 - 1.17	0.32
Year*Season interaction^d^	2001*spring		0.58	0.30 - 1.12	0.11
	2001*summer		**2.98**	**1.51 - 5.86**	**0.002**
	2001*winter		0.66	0.35 - 1.25	0.20
	2002*spring		0.95	0.51 - 1.76	0.86
	2002*summer		0.97	0.46 - 2.01	0.93
	2002*winter		1.34	0.72 - 2.49	0.35
	2003*spring		0.77	0.44 - 1.36	0.37
	2003*summer		0.66	0.34 - 1.31	0.23
	2003*winter		**2.06**	**1.18 - 3.06**	**0.01**
	2004*spring		0.85	0.51 - 1.40	0.52
	2004*summer		1.22	0.69 - 2.17	0.49
	2004*winter		**2.24**	**1.34 - 3.74**	**0.002**
	2005*spring		**2.19**	**1.39 - 3.47**	**0.001**
	2005*summer		**2.06**	**1.23 - 3.48**	**0.006**
	2005*winter		0.98	0.61 - 1.59	0.94
	2006*spring		**1.61**	**1.01 - 2.57**	**0.045**
	2006*summer		1.09	0.65 - 1.85	0.74
	2006*winter		1.57	0.97 - 2.54	0.07
	2007*spring		0.93	0.59 - 1.45	0.74

a. Logistic regression using generalized linear model ML estimation with the logit link, binomial distribution and total number of PRRSV PCR tests ordered representing the denominator.

b. Odds ratio.

c. Suspected *a priori* may be a confounding variable.

d. Interaction term.

bold text – significant variables.

## Discussion

The key finding of this study is that the weekly probability of PRRSV PCR positivity at the AHL decreased during the Ontario PCVAD outbreak. Thus, the results of PRRSV PCRs generated through laboratory test requests are an untapped source of swine health data that could be monitored for heightened swine disease outbreak awareness. A large proportion of negative test results do not specifically identify the novel disease or disease pathogen. However, monitoring the trends of such negative results could provide an early indication of disease diagnostic dilemmas occurring in the field. In other words, monitoring the results of such first-order tests could be used as an early indicator of a disease outbreak, a form of syndromic data. This could improve the recognition of a novel outbreak without having to wait the extra time it takes to reach a definitive laboratory diagnosis through the use of follow-up tests. For the Ontario swine industry this could have beneficial implications for the timely detection of swine disease outbreaks and with identifying and utilizing novel data sources for such timely detection [20].

The decrease in the weekly probability of positive PRRSV PCR results during the PCVAD outbreak could be extrapolated to suggest that practicing veterinarians were attempting to diagnose a new disease or syndrome (i.e., the PCVAD outbreak) by initially investigating for the presence of PRRSV through the use of the PRRSV PCR. Hence, monitoring PRRSV PCR requests, and more importantly, the results from these tests, has the potential to represent what veterinarians face in the field with respect to disease diagnosis. The PRRSV PCR used at the AHL did not change until after the PCVAD outbreak was resolved indicating that the changes in test positivity were not a result of changing test accuracy.

The results for the PRRSV ELISA model were not associated with the PCVAD outbreak likely due to data management issues identified in the study. Case submissions were not always clearly identified as to whether they were for monitoring or diagnostic purposes. Consequently, some submissions misclassified as diagnostic submissions were actually associated with routine farm monitoring and not part of a disease investigation. During the initial data management process, case submissions and test requests associated with semen specimens were dropped, as they were felt to represent herd monitoring for PRRSV instead of a disease investigation process. The ELISA, however, uses a serum sample that detects antibody, whereas the PCR, that detects antigen, is routinely performed on semen for boar stud herd PRRSV monitoring. Consequently, when cases associated with semen specimens were excluded, the PRRSV PCR data was likely more representative of true diagnostic cases versus monitoring cases.

The results of the PRRSV ELISA model may have also been influenced by many herd management and demographic changes that occurred in the Ontario swine industry during the study period. The number of total hogs in the province grew and the industry consolidated, with farms becoming larger and more species specialized [21]. With larger herd size came an increased awareness that disease outbreaks in larger herds have greater potential to create more severe mortality, morbidity, and economic consequences. Hence, management practices changed with respect to an increased understanding of the need for herd monitoring through testing [22]. For example, many on-farm PRRSV monitoring and eradication strategies were employed with the most common being the “test and removal” and the “herd closure and rollover” techniques [22]. Both involve frequent PRRSV testing of clinically normal animals.

This study highlights the importance of data quality at the time of collection. Mandatory field requirements on laboratory submission forms, such as those used by Gibbens et al., (2008), could improve upon the classification of monitoring versus diagnostic type cases [6]. In this study, case submission demographic information associated with the laboratory submissions was incomplete. For example, the age of the pigs being tested was not consistently recorded, and total animals-at-risk for a submission was often omitted. Additionally, the test requests and associated results could not be extracted from the database system together. This created considerable manual manipulation of the data to generate files that contained both the tests requested and associated results. The AHL currently has a new data management system in place with the main objective to improve disease surveillance activities and the utilization of such data.

The AHL is the largest veterinary diagnostic laboratory in Ontario and is the predominant laboratory used for swine diagnostics by practicing veterinarians in the province [16]. The Ontario Ministry of Agriculture and Rural Affairs (OMAFRA) partially funds food-animal producers/clients for certain testing at the AHL, which acts as an incentive for veterinarians and producers to use the services of the AHL. Consequently, data generated by the results of test requests at the AHL is believed to represent a large proportion of the diagnostic testing for the Ontario swine industry. Extrapolation beyond the Ontario swine industry should bear this in mind when considering inference to other swine populations. Use and interpretation of data from the diagnostic laboratory or laboratories representing the bulk of testing for the swine population under surveillance should be considered. Amalgamation of PRRSV PCR data from multiple diagnostic laboratories would have to ensure that the PRRSV PCRs were validated between laboratories [10].

A bias that presents itself in this study, as well as other studies using laboratory- derived data for disease surveillance purposes, is that the pigs being tested by the AHL represent farms/producers that seek veterinary professional services. While a large proportion of herds in Ontario probably seek the advice of veterinarians [23] and the services of the AHL, the exact proportion of producers conducting and not conducting PRRSV testing during the study period was unknown.

Future studies should employ surveillance monitoring and statistical tools to further investigate the usefulness of monitoring counts or clusters of negative test results. The application of cumulative sum-based (CUSUM) methods and other cluster detection techniques, such as the scan statistic, to the count or proportion of tests results should be considered [8,24]. The inclusion of such techniques in a disease surveillance system could present the diagnostic laboratory with a unique opportunity to play a central role in communicating disease trends to practitioners. Increased and timely communication of test results to veterinarians and other interested stakeholders might raise awareness of a disease outbreak, stimulate further discussion and dialogue, and pool knowledge and resources regarding potential disease concerns or outbreaks occurring in the field.

## Conclusions

This study showed that during the PCVAD outbreak in Ontario from December 2004 - May 2006, the probability of a positive PRRSV PCR at the AHL decreased. We conclude that when an increase in negative test results occurs (or decrease in positivity) it suggests that a new disease agent may be emerging in the population. PRRSV ELISA positivity did not yield a similar significant association possibility due to incomplete information associated with the test request at the time of submission. The results of this study support the importance of practitioners providing accurate and complete demographic and clinical history information on submission forms when requesting tests from a diagnostic laboratory.

Future research initiatives should be focused on CUSUM-based and cluster detection techniques for outbreak detection using the results of test requests made by veterinarians to diagnostic laboratories. To the authors’ knowledge this is the first study to document how a novel swine disease outbreak influenced the results of PRRSV tests requested by veterinarians.

## Competing interest

The authors declare no conflict of interest.

## Authors’ contributions

All authors were involved with the analysis and the interpretation of the data and with the revising of the manuscript critically for intellectual content. TO performed the cleaning and manipulation of the data, statistical analysis, and drafting and revising of the manuscript. All authors read and approved the final manuscript.
